# The Minima of Viscosities

**DOI:** 10.3390/ma17081822

**Published:** 2024-04-16

**Authors:** Michael I. Ojovan, Dmitri V. Louzguine-Luzgin

**Affiliations:** 1 Department of Materials, Imperial College London, South Kensington Campus, Exhibition Road, London SW7 2AZ, UK; 2 Department of Materials Science and Engineering, The University of Sheffield, Sir Robert Hadfield Building, Mappin Street, Sheffield S1 3JD, UK; 3 Advanced Institute for Materials Research (WPI-AIMR), Tohoku University, Aoba-Ku, Sendai 980-8577, Japan; dml@wpi-aimr.tohoku.ac.jp; 4 MathAM-OIL, National Institute of Advanced Industrial Science and Technology (AIST), Sendai 980-8577, Japan

**Keywords:** viscosity equation, minimal viscosity, bare mass, effective mass

## Abstract

The Trachenko–Brazhkin equation of the minimal possible viscosity is analysed, emphasising its validity by the account of multibody interactions between flowing species through some effective masses replacing their true (bare) masses. Pressure affects the effective masses, decreasing them and shifting the minimal viscosity and the temperature at which it is attained to higher values. The analysis shows that effective masses in the Trachenko–Brazhkin equation are typically lighter compared bare masses; e.g., for tin (Sn) the effective mass is m = 0.21m_Sn_, whereas for supercritical argon (Ar), it changes from m = 0.165m_Ar_ to m = 0.129m_Ar_ at the pressures of 20 and 100 MPa, respectively.

## 1. Introduction

Viscosity quantifies the resistance of material to flow and indicates the ability to dissipate momentum, arising because of a transfer of momentum between fluid layers moving at different velocities. For glass-forming liquids, viscosity changes by many orders of magnitude, attaining values exceeding 10^14^–10^12^ Pa⋅s in the vitreous state and decreasing to less than 10 Pa·s at the melting temperature. Indeed, in glass technology, the so-called strain point below which fracture will occur before the onset of plastic deformation corresponds to the temperature at which the viscosity is 3 × 10^13^ Pa·s, with the glass transition temperature typically being near it. The conventional melting point for glass which does not crystallise on heating corresponds to the temperature at which the viscosity is 10 Pa·s, so that the melt is fluid enough to be considered a liquid. Generically, the viscosity of matter has a minimal possible value which is revealed by available viscosity equations applied in the high temperature range of melts such as the double-exponent equation [1,2,3], also termed the Douglas–Doremus–Ojovan (DDO) model [4] or the Eyring and Kaptay (EK) models [5,6]. Moreover, recently, Trachenko and Brazhkin analysed viscous flow, deriving a universal equation for the minimum viscosities of materials [7] by merging two asymptotic cases, that of a liquid and a gas. This enabled an approximate (within order of magnitude) estimation of the minimal viscosity, with the observation that it is not significantly spread, at least by its order of magnitude. This equation resulted in further investigations, particularly for metallic systems [8,9,10], although the minima or viscosity were discussed priorly for organics based on condensed matter approaches rather than the merge of two asymptotic behaviours of matter [11]. The Trachenko–Brazhkin (TB) equation of minimal viscosity, η_min_ contains only basic universal constants (mass of electron m_e_ and Plank constant, ħ, in combination with the density of matter, ρ, and mass of flowing material species (atoms or molecules), m:

η_min_ = ρħ/4π(m_e_m)^1/2^, (1)

For the kinematic viscosity ν_min_ = η_min_/ρ = ħ/4π(m_e_m)^1/2^, the TB analysis shows that only fundamental constants (ħ and m_e_) and the mass of the atom or molecule (m) are contained, a fact which has allowed for TB to coin the notion of “elementary” viscosity [7]. The TB analysis gives minimal viscosity values of the order of 0.1 mPa·s [7,8,9], although it does not determine the crossover temperature when the viscosity is minimal, and it is generically provided via (1) rather rough numerical estimations, with some exceptions. Regarding the inaccuracy of the results obtained using (1), we assume that this is because of the non-exact fit within the merging process of the condensed matter asymptote equations of viscosity, such as that used before in [11] with one valid for gases. Such inaccuracies arise while applying the same effective masses of flowing units (species which are atoms, molecules, and clusters depending on the substance), for both the gaseous phase and condensed matter, a procedure which does not account for many-body interactions characteristic of liquids and practically absent in gases. One should note that the minimum of viscosity connected with the liquid-to-gas phase transition is rather expected. However, even in supercritical liquids where boiling is suppressed, the minimum viscosity is also achieved before entering into the plasma state [12,13].

The purpose of this communication is to align a pathway of utilisation of TB Equation (1) enabling the estimation of minimal viscosities to numerical data being equal or close to that found from experimental works. We argue that the mass, m, to be used in Equation (1) is not the bare atomic mass of moving atoms or molecules but rather some effective masses which, depending on the system parameters, are modified to account for the multi-body interactions between them. This substitution arises from the analysis of viscous flow based on the EK model [5,6] and configuron concept which follows the Mott [14] and Doremus [2] approaches, whereby the flow of condensed matter is treated as being facilitated by structural defects in the form of broken chemical bonds termed configurons in the case of multi-body systems such as condensed matter or superfluid matter.

## 2. Minimal Viscosity from the Condensed Matter Approach

The Kaptay equation of viscosity [6] is based on the Eyring work [5] and is a quantitative description tested for many molten metallic systems [6,15]. It can be represented as follows:

(2) $$\eta (T)=A\frac{M^{1/2}T^{1/2}}{V^{2/3}}exp(\frac{E_{a}}{RT}),$$

where M and V are the atomic mass and molar volume, E_a_ is the activation energy, R is the gas constant, and T is the temperature. Equation (2) was tested on 101 measured points of 15 selected liquid metals, and the average value of constant A was found to be A = (1.80 ± 0.39) × 10^−8^ (J/Kmol^1/3^)^1/2^, while the activation energy E_a_ = B × RT_m_ where T_m_ is the melting point and the constant B = 2.34 ± 0.20 [6]. Similarly to the DDO model [11], Equation (2) has a well-defined minimum attained at the crossover temperature, T_vm_, provided by formula (3):

(3) $$T_{vm}=\frac{{2E}_{a}}{R}$$

and given by expression (4):

(4) $$\eta_{min}=A\frac{M^{1/2}({{2e}_{n}E_{a})}^{1/2}}{(R{)}^{1/2}V^{2/3}}$$

Here, e_n_ is the Euler number (e_n_ = 2.71828…).

The analysis of viscosity changes at high temperatures contains attempts to predict minimal viscosities, applying both TB Equation (1) [8,16] and Kaptay Equation (2) [15]; however, experimental confirmations of any such type of assessments were not the case until recent works by Patouillet and Delacroix who experimentally analysed the viscosity of melts up to very high temperatures, especially for tin (Sn) and lead (Pb) [17]. The measurements of high-temperature viscosity of tin [17] confirmed the occurrence of minimal viscosity η_min_ = 0.53 mPa·s at T_vm_ = 1600 °C not far from that derived in [15] and quite close to that calculated using Equations (3) and (4) (see, e.g., for additional details and discussion Ref. [10]), although the authors of [17] have not related their observations with the minimal viscosity predicted theoretically.

**Figure 1:**
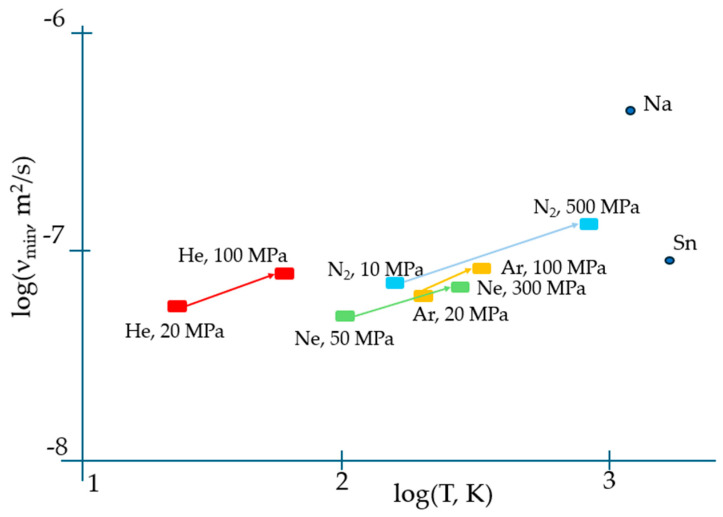
The effect of pressure on temperatures when the viscosity is minimal, and on kinematic viscosity minima, demonstrating their significant shift to higher values with pressure (upward and righthand), which can be interpreted in terms of the change of activation energy of flow, E_a_ (see Equations (3) and (4)). The minimum kinematic viscosities of Sn and Na at a normal pressure are also indicated with data taken from [17] and [18], respectively.

Apart from correctly predicting the temperature when the viscosity reaches its minimum, Equation (2) results in an expression for η_min_ which contains both individual atomic characteristics (M, V) and the activation energy of flow E_a_ which is a distinct collective parameter of the system of atoms/molecules rather than of an individual specie alike occurring in the gaseous phase when the interaction between them can be fully neglected. Moreover, the temperature when the viscosity attains its minimum given by Equation (3) is explicitly dependent on the collective parameter of the system, i.e., E_a_. This suggests that most probably, the universal TB equation of viscosity minimum needs amendments to properly describe the experiment. The latter evidently indicates that the minimum viscosity of matter strongly depends on the pressure. Figure 1 illustrates this dependence for the materials analysed in [7].

**Figure 2:**
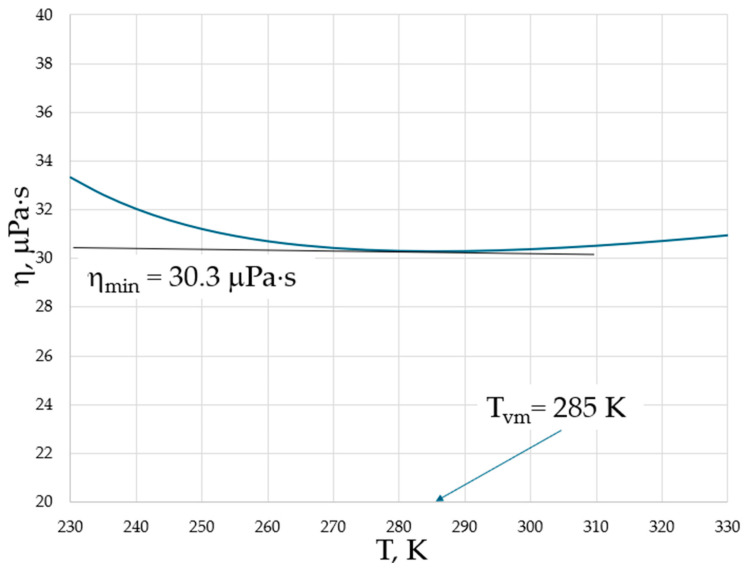
The dynamic viscosity of Ar at a 20 MPa pressure with data taken from the NIST Chemistry WebBook [19].

Both higher ν_min_ and T_vm_ at higher pressures provide evidence for the importance of collective effects in reaching the minimal viscosity. However, in contrast to the logarithmic scale used in Figure 1, temperature is typically plotted on a linear scale in experiments; therefore, the minima of viscosities are on a linear scale of temperature, and the exact determination of temperature when they occur is not straightforward (see, e.g., Figure 9 of [17]). We show this explicitly in Figure 2 for the temperature dependence of viscosity of argon (Ar) at a 20 MPa pressure based on data from the NIST Chemistry WebBook [19], demonstrating that the curves are smooth with a shallow minimum, and that the exact determination of the minimum position is difficult even in the case of using the same linear scale for both viscosity and temperature.

**Figure 3:**
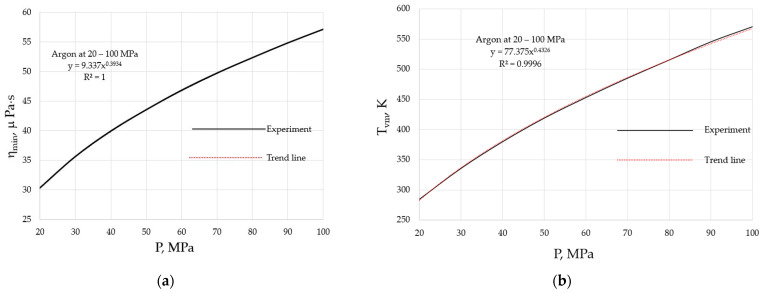
Pressure dependencies of the minimal viscosity and the crossover temperature when the minimum is achieved: (**a**) minimal viscosity, η_min_; (**b**) crossover temperature, T_vm_. Viscosity data are taken from the NIST Chemistry WebBook [19].

Both the η_min_ and T_vm_ of materials are pressure-dependent, as they follow from Equations (2) and (3), and are illustrated in Figure 3 for argon at the pressures of 20 to 100 MPa.

**Figure 4:**
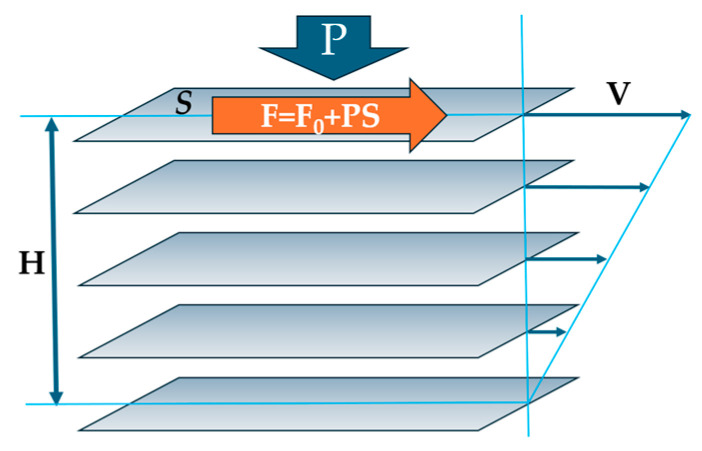
Schematic of imaginary moving layers in a flowing liquid.

The approach within the configuron percolation theory (CPT) accounting for the breaking of chemical bonds which facilitate shear flow has led to a two-exponential form of viscosity as a function of temperature: the DDO model, which is, as a rule, providing a better fitting to experimental data compared to single-exponential expressions [3,4,11]. The activation energy of flow at high temperatures within CPT is constant and low, being equal to the enthalpy of motion of configuron E_a_ = H_m_ [3,11]. The H_m_ in glasses can be considered an elastic strain energy or friction between moving layers since, at the microscopic level, the viscosity arises due to the transfer of momentum between fluid layers moving at different velocities, as explained in Maxwell’s kinetic theory (Figure 4).

The shear viscosity coefficient, η, by definition is the ratio of shear stress divided to the velocity gradient: η = (F/S)(V/H), where F is the force applied to the surface of area S, V is the velocity, and H is the separation height between the two layers (see Figure 4). The tighter the bound layers and the more difficult their motion, the higher the resulting viscosity. The external pressure will push layers against each other, therefore increasing the force of internal friction between the layers, hence F = F_0_ + PS, where the intrinsic force F_0_ is related to the surface tension of matter σ via friction coefficient μ: F_0_ = *a*μσ with *a* being the proportionality coefficient. We can thus assess the H_m_ assuming that the internal friction between the imaginable layers of flowing liquid is caused by the existing bonding between them without any external pressure (H_m0_), with an added part proportionally dependent on the applied external pressure, P, which accounts for the fact that the higher pressure, the higher the force pressing the layers against each other:

(5) $$H_{m}=H_{m0}+b\mu P^{\alpha }$$

where α is the power coefficient and *b* is the proportionality coefficient (in m^3^/mol). Thus, at higher pressures, H_m_ increases in line with Frenkel’s original ideas that loose molecular cages facilitate the escape of atoms from cages often used in practice [20,21,22,23,24], whereas oppositely to that, a compressed molecular cage will prevent the atoms to escape from it. Hence, in the first approximation, if H_m0_ >> *b*μP^α^, for the crossover temperature and the viscosity minimum, the following functions hold:

(6) $$T_{vm}=A_{P}+B_{P}P^{\alpha },\;\eta_{min}=C_{P}+D_{P}P^{\alpha },$$

where A_P_ = 2H_m0_/R, B_P_ = 2*b*μ/R, C_P_ = AM^1/2^(2e_n_)^1/2^/R^1/2^V^2/3^, and D_P_ = AM^1/2^(2e_n_)^1/2^A_P_/2R^1/2^V^2/3^B_P_. The higher the external pressure, the higher E_a_, which naturally explains the pressure shifts of both the minimal viscosity, as seen in Figure 3a, and the crossover temperature, as seen in Figure 3b. From these data, it follows that α ≈ 0.4.

## 3. Effective Masses of Flowing Units

At a first glance of the above results, we may conclude that the collective effects are fully missed in TB Equation (1). Nevertheless, we are prone to dismiss such conclusions with arguments as follows. Apart from fluid density, ρ, and the mass of flowing unit, m, Equation (1) contains fundamental constants only and shall therefore be augmented with a fitting coefficient effectively dependent on the type of material aiming to correctly describe the experiment. As seen (see Figure 1), the kinematic viscosity is also affected by pressure; thus, an augmenting fitting coefficient shall also depend on pressure, from which it follows that the coefficient is clearly not a universal constant missed within the merging analysis. However, the TB equation contains the mass of flowing unit m, which, in the condensed matter, is not necessarily equal to that of a single atom or molecule. Such discrepancies are well known in calculating the effective sizes of flowing units in liquids (see Table IV of Ref. [2]), which shows rather small sizes compared with the actual sizes of molecules. The situation is similar to that encountered in conductors when the mass of charge carriers is not necessarily equal to m_e_ for an electronic or ionic mass for ionic conductors for the reason of multibody interactions in the system [25,26] where even negative effective masses can appear [27]. Hence, the mass of flowing unit in the TB equation should be an effective mass m rather than the bare mass of an atom or molecule of flowing substance m_i_:

(7) $$m=M_{rel}m_{i}$$

where M_rel_ is the coefficient which accounts for the multibody interaction in the system of flowing units and m_i_ = M/N_A_, where N_A_ is the Avogadro number. Effective masses of flowing units can be derived from data on known minimal viscosities, η_min,exper_, using Equation (7):

(8) $$m={\frac{1}{m_{e}}\left(\frac{\rho \hbar }{4\pi \eta_{min,exper}}\right)}^{2}$$

Coefficients M_rel_, which can be also termed the relative masses of lowing units (as M_rel_ = m/m_i_), can be found through the comparison of minimal viscosity from the experiment η_min,exper_ with η_min,TB_ available from the calculation while using TB Equation (1) with bare masses m_i_ instead of effective masses m (see, e.g., [7,8,9]):

(9) $$M_{rel}={\left(\frac{\eta_{min,TB}}{\eta_{min,exper}}\right)}^{2}$$

Table 1 shows the effective masses of flowing units M_rel_ of some materials analysed in Tables 1 and 10 of references [7] and [17].

**Figure 5:**
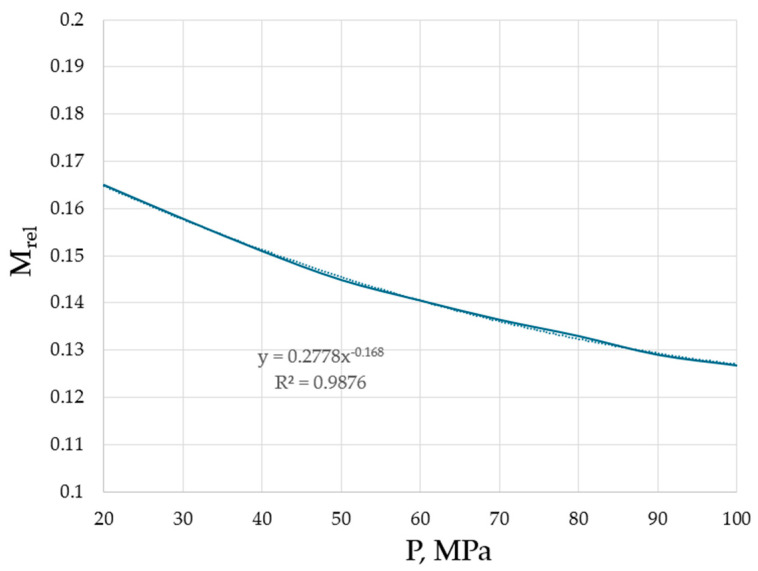
Effective masses of the flowing units in argon as a function of pressure. Minimal viscosities were taken from the NIST Chemistry WebBook [19].

Hence, it is revealed that the effective masses are typically, although not necessarily smaller compared to the bare masses of species. However, it is unambiguously revealed that the higher the pressure, the lower the effective masses of the flowing units. This trend is explicitly shown in Figure 5 for argon, the interest in structural changes of which, at varying pressures, is not diminishing, demonstrating the existence of crystalline clusters within the background of an amorphous phase, even at ultra-fast cooling rates [28].

The trend from both Table 1 and Figure 5 is evident: the higher the pressure, the closer the atoms and molecules to each other (see Figure 4), the stronger the interactions between them which lead to changes in effective masses, namely the decrease in effective masses compared to the bare masses of flowing units (atoms, molecules, clusters). Although the TB equation could be corrected with a non-accounted coefficient which will change the numerical values of M_eff_, the trend will be preserved: the higher the pressure, the lower the effective mass of the flowing unit.

## 4. Discussion

Having a dynamic topologically disordered structure, liquids are typically assumed with uniformly distributed species (atoms, molecules), although this is true only macroscopically and on average observation time, whereas at the microscopic scale and short observation times (nanoscale), they are dynamically inhomogeneous media. Indeed, due to the relatively high density of liquids, which is close to that of solids, metastable clusters made up of several or more species are always formed because of close (almost solid state) distances between species and the tendency to form microscopically metastable collective structures (clusters) which diminish the energy of the system. Some clusters can, with time, disintegrate, but the lower the temperature, the larger the sizes and longer the lifetime of clusters [13,15,29,30,31]. Typical examples of ordering in liquids are tetrahedral structures in silica melts and icosahedral structures in molten metallic glasses [31,32,33,34] the assemblage of which, on the lowering of temperature, leads to either crystallisation or vitrification (liquid–glass transition) [35,36,37,38,39,40]. The existence of both persistent (static) and dynamic structures was recently demonstrated, e.g., in [34], where static structural features included the peak positions of the structure factor and pair distribution function, bond angle distribution, number of tetrahedra, cluster connection, free volume, and dynamical structural features comprising the relaxation time, diffusion coefficient, and non-Gaussian parameter. Long-wavelength density fluctuations (Fischer clusters) revealed the occurrence of local ordering in glass-forming liquids [41,42]. It was also shown that the density fluctuations have correlation lengths of up to 300 nm and are characterised by a fractal structure with the dimension D < 3 [43]. Moreover, within CPT, the liquid state is viewed as consisting of macroscopic clusters with a fractal geometry surrounded by macroscopic configuron clusters, and the glass transition as a percolation via broken bonds being formally is a two-state model in which the high energy level is the broken bond and the low one is the intact bond, operating on a bond lattice instead of the more conventional particle lattice [3,38,39].

It is important to emphasise that the tendency to cluster is observed in liquids above the melting temperature (liquidus), e.g., the atomic structure of melts of simple metals demonstrates that the dense part of the liquid in molecular dynamic models represents branched chains of almost regular tetrahedra linked in pairs by faces, with the resulting clusters being fractal with the dimension D = 2.6 [44]. The occurrence of local ordering in liquids is confirmed experimentally in many works, e.g., [45,46,47]. Moreover, the memory effects of thermal evolution were found above the liquidus temperatures of silicate systems [48]. Recent analysis has shown that the de-wetting temperatures of pre-frozen (pre-solidified) and grafted layers in ultrathin films can be viewed as the melt memory effects lead to new scenarios of crystallisation [49]. These observations overturn simplified notions that the structure of a liquid above the liquidus temperature is completely disordered and has neither a long-range nor middle-range order. Hence, liquids are homogeneous and fully disordered at large times, exceeding the lifetime of metastable formations and spatial scales large enough and being characterized by a short- and medium-range order locally at distances of the size of molecules and metastable clusters. At smaller scales, liquids are neither completely homogeneous nor fully disordered where ordering does not mean periodicity, as quasicrystals being ordered are not periodic. This shall be accounted for in the description of the liquid properties observed, including the viscous flow and the viscosity, because even for dilute systems, clusters are formed arising from the direct contact aggregation of particles [50].

**Figure 6:**
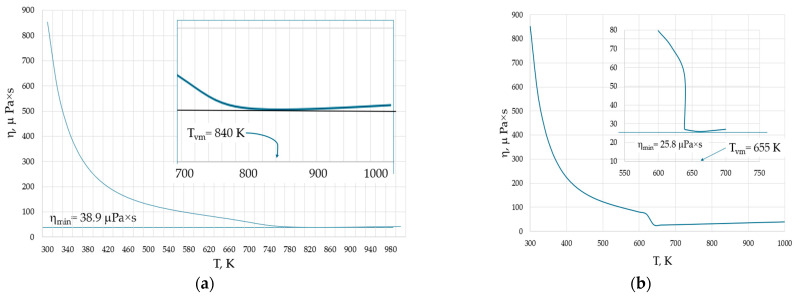
Isobaric viscosities of water: (**a**) the viscosity at P = 50 MPa. At temperatures exceeding 660 K, the water becomes supercritical and the viscosity minimum occurs at T_vm_ = 840 K in the supercritical state. (**b**) The viscosity at P = 20 MPa. At temperatures exceeding 638.9 K, the water becomes gas and the viscosity minimum occurs at T_vm_ = 655 K in the gaseous state. The behaviour of viscosity near their minimal values is shown by the insets. Viscosity data are taken from the NIST Chemistry WebBook [19].

Inhomogeneities observed in liquids and gases also occur in the supercritical state [13,51]. For example, the structure of pure supercritical water (H_2_O) exhibits both tetrahedral liquid-like and gas-like configurations depending on the external pressure [51]. The supercritical state of water is observed at temperatures and pressures exceeding 647 K and 22 MPa, respectively. Figure 6 shows the temperature dependence of the viscosity of water at two pressures: (a) 50 MPa, when it becomes supercritical above 660 K; and (b) at 20 MPa, when it becomes gaseous above 638.9 K.

The higher the pressure, the higher both η_min_ and T_vm_, in line with Equation (6). However, an interesting observation is that at both pressures, the minimal viscosity is not attained in the liquid phase of water. Instead, the viscosity is minimal either in supercritical or gaseous water. In the latter case, this seems to be surprising, as the equations of the viscosity of gases have no minima and the viscosity should always grow with temperature.

Concerning metallic systems, TB Equation (1) with bare masses of metals works only within the orders of magnitude range of viscosity and is not resulting in the description of the experiment which shows that metallic alloys differ by viscosity minima by an order of magnitude. The TB equation gives a narrow range within 23% difference only, e.g., within (2.04–2.66) × 10^−8^ m^2^/s, as seen in Table 1 of Ref. [8]. An account for effective instead of the bare masses of species allows for an exact description of experiment. An important aspect for metals is either that the T_vm_ is below or above the critical temperature T_c_. Table 2 compares the T_vm_ calculated in [10] with the boiling point temperatures T_b_ [52] and critical temperatures T_c_ estimated from sound velocities applying the isochoric thermal pressure coefficient method [53].

Hence, Table 2 reveals that all metals will exhibit minimal viscosities, far below that required to achieve the critical state. Thus, the experimental determination of T_vm_ is experimentally achievable using a conventional viscometer, which may be of interest for the practical applications of metallic coolants of fast breeder reactors [18]. Earlier analysis of viscosity of sodium and other alkali metals have confirmed the existence of achievable minima of viscosity [55,56]. The most recent large-scale molecular dynamics simulations performed for LiF and Pb also confirmed the lower bound of the viscosity in the liquid state [57]. The experimental data of viscosities analysed in [7] and used to support the conclusion on the existence of minimal viscosities were taken for pressures far above the critical pressure. This was performed aiming to avoid crossing the boiling line where substances undergo the liquid–gas transition accompanied by a sharp change of viscosity, although this was not necessarily for the generic theoretical conclusion on the existence of minimal viscosity. However, data from column 2 of Table 2 show that the minimal viscosities of metals can be achieved at temperatures below not only the critical temperatures, but also below their boiling points, at least for Hg, Bi, Ga, and Sn. This is not the case for Pb, the viscosity of which was analysed at temperatures up to 1770 [17] without detecting any shallow minimum in line with the data of [10]. This, however, was the case for Sn, for which the experiment demonstrated that T_vm_ = 1600 °C and η_min_ = 0.53 mPa·s, being relatively close to the theoretical parameters obtained using the EK viscosity model (Equations (3) and (4)): T_vm_ = 1447 °C and η_min_ = 1.1 mPa·s. Data for the other metallic systems are provided in Table 1 of [10]. Thus, we may infer that metallic systems, and particularly Hg, Bi, Ga, and Sn, indeed present a high potential for future experimental work to further validate and refine the above-proposed modifications to the TB equation.

We have calculated the effective masses of the flowing units by comparing the available experimental data (mainly from Ref. [19]) with that obtained using TB Equation (1) [7]. Only in this case can we infer that typically M_rel_ < 1 (see the last column of Table 1). However, it is generically possible that TB Equation (1) contains a non-accounted numerical coefficient C_N_ which can expectedly be a large, compared unit, and Equation (1) will instead read η_min_ = C_N_ρħ/4π(m_e_m)^1/2^. The method used in [7] to derive equation (1) resembles the well-known similarity and dimensional methods widely used in the past especially for solving a wide range of problems in the mechanics and hydrodynamics such as that characteristic for the theory of explosions and in the astrophysics [58]. These methods cannot however derive the exact values of numerical coefficients in equations derived which can be quite large (e.g. the coefficient C_N_ = π or let say C_N_ = 16π^2^) meaning that the effective mass (9) would differ by several orders of magnitude (e.g. one or four orders in the above two examples) from that given by Equation (10). In this case, the masses of the flowing units could be in liquids not smaller than the individual masses of species m_i_ because in this case, the relative masses will be given by

(10) $$M_{rel}={\left(C_{N}\right)}^{2}{\left(\frac{\eta_{min,TB}}{\eta_{min,exper}}\right)}^{2}$$

where C_N_ > 1. Nevertheless, even in this case, the trend seen in Table 1 and Figure 5 will be preserved, indicating that the higher the pressure, the lower the effective mass of a flowing unit. This in turn indicates the increasing role of the interactions between particles in the collective parameters of systems, one of which is the effective mass of the moving units in fluids. The effective masses are hence sensitive not only to the characteristics of the substances analysed, but also to external conditions, namely external pressure. The extreme increase in temperature can additionally cause the dissociation of molecules and ionisation, and leads to the transformation of matter to a plasma state, with possible intermediate condensed phases such as the condensed Rydberg matter, the rheological behaviour of which is unknown [59,60,61,62], although these can be of practical use to validate the TB approach to viscosity and its modification, applying the above concept of effective masses.

Finally, we note that the Eyring–Kaptay (EK) equation used here to find the temperature T_vm_ was extended from the temperature range, where its correctness was confirmed by the experiment [6,63], to the range where its validity may be questionable; therefore, Equation (3) is an approximate assessment of the crossover temperature T_vm_. Accounting for the fact that the DDO model of viscosity gives a twice-smaller value of T_vm_ = E_a_/R [10,11], we can admit that in the experiment, the viscosity minima could be expected to be within the interval from E_a_/R to 2E_a_/R, in any case being directly proportional to the activation energy of fluid flow at high temperatures. As shown in Table 2, the minimum viscosity temperature is a fraction of the boiling point temperature for some metals, and above it for others (and would always be below TB within the DDO viscosity model). Therefore, experimental verification of the existence of minimal viscosities is possible, especially for metals within the reach of common viscometers.

## 5. Conclusions

The viscosity minima occur at crossover temperatures, T_vm_, which are considerably above the melting points and are universally observed in the liquid, gaseous, and supercritical states. An assessment of the crossover temperatures when the minima are observed is provided by Equation (3), with the minimal viscosity estimations given by Equation (4). These equations were derived extending the Eyring–Kaptay model of viscosity to a higher range of temperatures. Both the crossover temperature and the minimal viscosity increase with the increase in external pressure have a dependence on the first approximation given by Equation (6). Comparison of experimental data with the Trachenko–Brazhkin universal equation for minimal viscosity (1) suggests that it can correctly describe the experiment, provided that the effective masses of the flowing units of materials are used in it instead of the bare masses of single atoms or molecules.

## Figures and Tables

**Table 1:** Relative masses of the flowing units (M_rel_) of materials at the minima of viscosity.

Substance	Reference for η_min,exper_	M_rel_
Neon (Ne) at 50 MPa	[7] ^1^	1.09
Neon (Ne) at 300 MPa	[7]	0.54
Helium (He) at 20 MPa	[7]	4.23
Helium (He) at 100 MPa	[7]	2.04
Nitrogen (N_2_) at 10 MPa	[7]	0.4
Nitrogen (N_2_) at 500 MPa	[7]	0.1
Hydrogen (H_2_) at 50 MPa	[7]	0.87
Oxygen (O_2_) at 30 MPa	[7]	0.26
Water (H_2_O) at 100 MPa	[7]	0.18
Carbon dioxide (CO_2_) at 30 MPa	[7]	0.16
Methane (CH_4_) at 20 MPa	[7]	0.24
Carbon monoxide (CO) at 30 MPa	[7]	0.28
Tin (Sn)	[17]	0.21
Sodium (Na)	[18]	0.07

^1^ See Table 1 of [7].

**Table 2:** Crossover temperatures at which the viscosity is minimal compared with the critical temperatures of metals ^1^.

Metal	*T_vm_*, K [10]	*T_b_*, K [52]	*T_c_*, K [53]
Hg	604	630	1982
Na	1260	1156	2447
K	1210	1032	2288
Pb	2510	2022	5573
Bi	1550	1837	4620 ^2^
Ga	920	2477	4282
Sn	1720	2875	4536

^1^ The DDO model of viscosity gives a twice-lower value [11]. ^2^ The critical temperature was taken from [54].

## Data Availability

Data are contained within the article.
